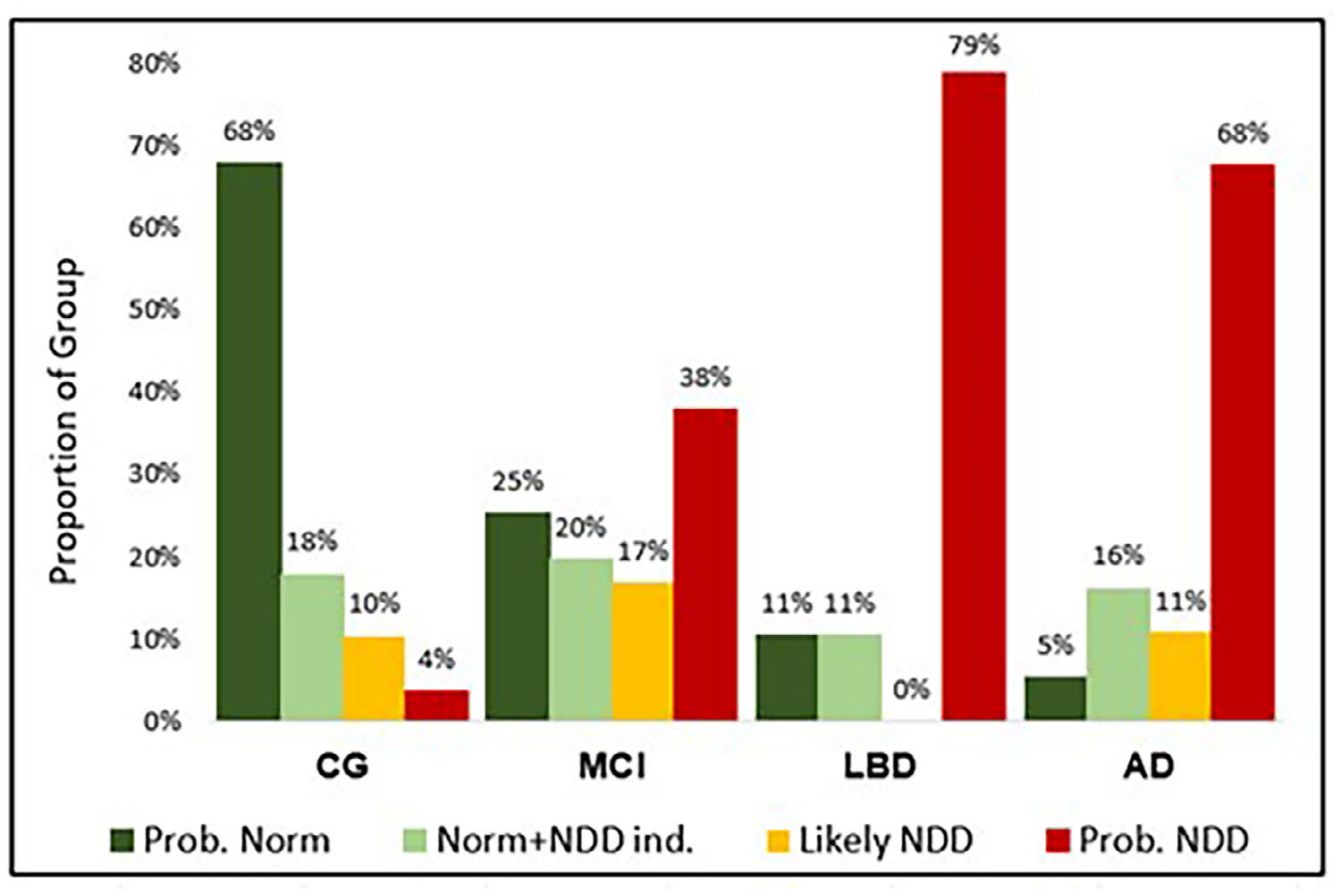# Sleep Biomarker‐Based Characterization of Neurodegenerative Disorder Phenotypical Risk

**DOI:** 10.1002/alz.089805

**Published:** 2025-01-09

**Authors:** Daniel J Levendowski, Christine M Walsh, Yeilim Cho, Debby W Tsuang, Joyce Lee‐Iannotti, Chris Berka, Brad Boeve, Thomas C. Neylan, Erik K St. Louis

**Affiliations:** ^1^ Advanced Brain Monitoring, Inc., Carlsbad, CA USA; ^2^ Memory and Aging Center, UCSF Weill Institute for Neurosciences, University of California San Francisco, San Francisco, CA USA; ^3^ VA Puget Sound Health Care System, Seattle, WA USA; ^4^ Department of Psychiatry and Behavioral Sciences, University of Washington, Seattle, WA USA; ^5^ Banner University Medical Center, Phoenix, AZ USA; ^6^ Mayo Clinic, Rochester, MN USA; ^7^ Department of Neurology, Mayo Clinic, Rochester, MN USA

## Abstract

**Background:**

In this pilot study, the diagnostic agreement for sleep biomarker‐based neurodegenerative disorder (NDD) risk probabilities was evaluated in patients with Alzheimer’s disease dementia (AD), Lewy body disease (LBD), mild cognitive impairment (MCI), and controls (CG) with a Mini‐Mental State Exam scores ≥28.

**Methods:**

Sleep biomarkers recorded with the Sleep Profiler (Advanced Brain Monitoring, Inc.) were used as inputs to a 4‐class machine learning algorithm trained to assign NDD risk probabilities to AD=27, LBD=19, isolated REM sleep behavior disorder=15, and CG=58. Input variables included age, time‐REM, non‐REM hypertonia, autonomic‐activation index, spindle‐duration, atypical‐N3, time‐supine, sleep‐efficiency, relative‐theta, and theta/alpha. Distributions of NDD risk were then assigned to AD=37, LBD=19, MCI=71, and CG=106 records.

Records with CG output probabilities ≥70% were labeled Probably‐Normal, and from 45‐70% Likely‐Normal with indications of a NDD added when the LBD, AD or prodromal synucleinopathy (pSYN) risks exceeded approximately 25%. Similar thresholds were used to assign Likely‐ or Probably‐ LBD, AD, pSYN, or Mixed based on the group probabilities. Likely‐Normal with indications consistent with the diagnosis trended toward agreement.

**Results:**

The proportions of each group with classifications consistent with the diagnosis or trended toward agreement were CG=86%, MCI=75%, LBD=79%, and AD=87%.

In the CG group, NDD risk assignments were Probably‐Normal=68% and Likely‐Normal with NDD indications=18%, Likely‐NDD=10%, and Probable‐NDD=4%.

The MCI group was assigned Probable‐NDD=38% (AD=26%, Mixed=10%, LBD/pSYN=2%), Likely‐NDD=17% (AD=7%, Mixed=6%, LBD=3%, pSYN=1%), Likely‐Normal with indications=20% (AD=9%, pSYN=7%, Mixed=4%) and Probably‐Normal=25%

The LBD group’s assigned risks consistent with the diagnoses included Probable‐NDD=74% and Likely‐Normal with pSYN indications=6% but included inconsistencies from Probable‐AD=5%, Likely‐Normal with AD indications=5%, and Probably‐Normal=11%.

AD group’s consistent risk classifications included Probable‐NDD=60%, Likely‐NDD=11%, Likely‐Normal with AD/Mixed indications=13% and countered by inconsistencies from Probable‐LBD/pSYN=8%, Likely‐Normal with pSYN indications=3%, and Probably‐Normal=5%.

**Conclusions:**

Consistencies between the diagnoses and assigned NDD risks agreements exceeded 75% in all four groups. Studies are underway to confirm the test‐retest reliability and/or evaluate NDD risk probabilities in other patient populations.

**Support**: NIH grants R44AG050326, R44AG054256, RO1AG060477, R33AG064271, ULRR024150, R34AG056639.